# Functional and Structural Characterization of ClC-1 and Na_v_1.4 Channels Resulting from *CLCN1* and *SCN4A* Mutations Identified Alone and Coexisting in Myotonic Patients

**DOI:** 10.3390/cells10020374

**Published:** 2021-02-11

**Authors:** Oscar Brenes, Raffaella Barbieri, Melissa Vásquez, Rebeca Vindas-Smith, Jeffrey Roig, Adarli Romero, Gerardo del Valle, Luis Bermúdez-Guzmán, Sara Bertelli, Michael Pusch, Fernando Morales

**Affiliations:** 1 Departamento de Fisiología, Escuela de Medicina, Universidad de Costa Rica, 11501 San José, Costa Rica; oscar.brenes_g@ucr.ac.cr; 2 Centro de Investigación en Neurociencias (CIN), Universidad de Costa Rica, 11501 San José, Costa Rica; 3 Istituto di Biofisica, CNR, 16149 Genova, Italy; raffaella.barbieri@ge.ibf.cnr.it (R.B.); sara.bertelli89@gmail.com (S.B.); 4 Instituto de Investigaciones en Salud (INISA), Universidad de Costa Rica, 11501 San José, Costa Rica; melissa.vasquez@ucr.ac.cr (M.V.); rebeca.vindas@ucr.ac.cr (R.V.-S.); jsroigf@yahoo.com (J.R.); 5 Escuela de Biología, Universidad de Costa Rica, 11501 San José, Costa Rica; adarli.romero@ucr.ac.cr; 6 Laboratorio de Neurofisiología (Neurolab), 11801 San José, Costa Rica; neurolab22@yahoo.com; 7 Sección de Genética y Biotecnología, Escuela de Biología, Universidad de Costa Rica, 11501 San José, Costa Rica; luis.enriqbg@gmail.com; 8 Scuola Internazionale Superiore di Studi Avanzati (SISSA), 34136 Trieste, Italy

**Keywords:** myotonia, chloride channel, sodium channel, *Xenopus* oocytes, electrophysiology, structure analysis

## Abstract

Non-dystrophic myotonias have been linked to loss-of-function mutations in the ClC-1 chloride channel or gain-of-function mutations in the Na_v_1.4 sodium channel. Here, we describe a family with members diagnosed with Thomsen’s disease. One novel mutation (p.W322*) in *CLCN1* and one undescribed mutation (p.R1463H) in *SCN4A* are segregating in this family. The *CLCN1*-p.W322* was also found in an unrelated family, in compound heterozygosity with the known *CLCN1*-p.G355R mutation. One reported mutation, *SCN4A*-p.T1313M, was found in a third family. Both *CLCN1* mutations exhibited loss-of-function: *CLCN1*-p.W322* probably leads to a non-viable truncated protein; for *CLCN1*-p.G355R, we predict structural damage, triggering important steric clashes. The *SCN4A*-p.R1463H produced a positive shift in the steady-state inactivation increasing window currents and a faster recovery from inactivation. These gain-of-function effects are probably due to a disruption of interaction R1463-D1356, which destabilizes the voltage sensor domain (VSD) IV and increases the flexibility of the S4-S5 linker. Finally, modelling suggested that the p.T1313M induces a strong decrease in protein flexibility on the III-IV linker. This study demonstrates that *CLCN1*-p.W322* and *SCN4A*-p.R1463H mutations can act alone or in combination as inducers of myotonia. Their co-segregation highlights the necessity for carrying out deep genetic analysis to provide accurate genetic counseling and management of patients.

## 1. Introduction

Non-dystrophic myotonias (NDM) are a group of hereditary muscle diseases characterized by myotonia, muscle stiffness and a non-dystrophic phenotype [1]. Myotonia, detected by electromyography (EMG), is primarily due to enhanced muscle excitability, leading to sustained bursts of discharges that correlate with the magnitude and duration of involuntary after-contractions [2,3]. Myotonia congenita (MC), either dominantly (Thomsen’s disease, dominant myotonia congenita (DMC)) or recessively (Becker generalized myotonia, recessive myotonia congenita (RMC)) inherited, paramyotonia congenita (PC, dominantly inherited) and the sodium channel myotonias (SCM, dominantly inherited) are NDM [4,5].

Mutations in the *CLCN1* and *SCN4A* voltage-gated ion channel genes, encoding the skeletal muscle chloride (ClC-1) and sodium α-subunit (Na_v_1.4) channels, respectively, are responsible for these diseases [6,7]. Most of the more than 200 *CLCN1* mutations trigger a loss-of-function, while *SCN4A* mutations (about 83 mutations) trigger a gain-of-function (http://www.hgmd.cf.ac.uk/ac/index.php) [2,8,9]. In order to characterize biophysical or biochemical alterations induced by presumably disease-causing mutations, and to confirm their causality, wild-type (WT) and mutated ion channels are usually expressed in heterologous systems and studied electrophysiologically under voltage-clamp conditions. A common expression system are *Xenopus* oocytes because they bear an exceptionally low background of endogenous voltage-gated channels [10,11]. A particular advantage of *Xenopus* oocytes compared to transfection-based approaches is that the single cell injection allows to control the stoichiometric amount of RNA delivered. This allows for a straightforward quantification of effects of mutants on current densities, comparing WT and mutant in the same batch of oocytes. This is even more important for multimeric channel complexes, for example, for dimeric ClC channels and transporters. Heterozygosity of mutant/WT can be quantitatively reproduced and possible dominant effects of mutants in the dimeric complex can be investigated [9,12,13].

In healthy skeletal muscle, ClC-1 is responsible for stabilization of the resting membrane potential [2,9] and contributes to the repolarization during action potential firing [2]. Instead, Na_v_1.4 is responsible for the initiation and propagation of the action potential [2,14]. Therefore, mutations in these genes have been recognized as the basis for enhanced muscle excitability seen in patients with NDM [2,15,16].

Here, we combined clinical, molecular, functional, and structural findings of new and reported but undescribed *CLCN1* and *SCN4A* mutations found in three Costa Rican families affected with NDM. This integral approach is expected to provide better understanding of the mutations related with these conditions.

## 2. Materials and Methods

### 2.1. Study Population

**Figure 1:**
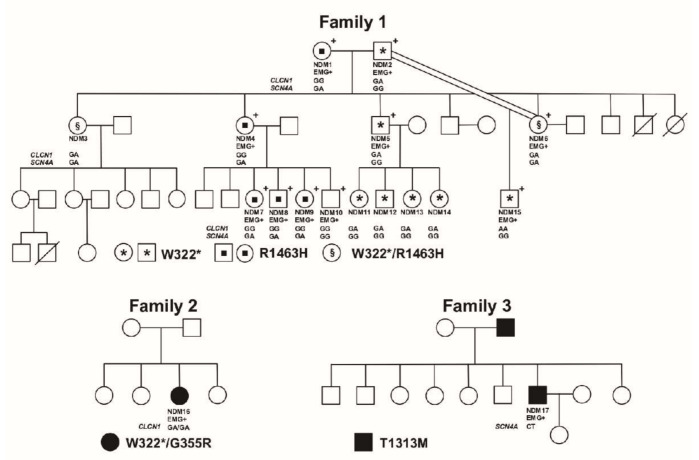
**Pedigrees of the three families segregating *CLCN1* and *SCN4A* mutations.** (Family 1) Clinical information for this family has been published already, including detailed information on which individuals were clinically evaluated and that resulted affected [13]. Coded (NDM1-15) family members were studied clinically and/or genetically. Circles represent females and squares represent males. Individuals marked with a + (upper right of the symbol) indicates that those individuals had been previously clinically evaluated [13]. Clinical and genetic information is indicated below each symbol. EMG+ indicates positive for myotonia in the electromyographic test. Genotype for each individual tested for the *CLCN1* and *SCN4A* mutations is also indicated below the symbol. For *CLCN1*, genotypes GA/AA indicate that the individual carries the W322* mutation, while genotype GG indicates that the individual is W322*-free. For *SCN4A*, genotype GA indicates that the individual carries the R1463H mutation, while genotype GG indicates that the individual is R1463H-free. The figure also shows the code to determine which patients carry both or just one of the mutations. Clinical and genetic information for the uncoded family members is unavailable. (Family 2) Individual NDM16 resulted carrier of two *CLCN1* mutations. Coded (NDM16) family member was studied clinically and/or genetically. Filled symbol indicates that the individual is symptomatic. Genotype for NDM16 for both mutations is indicated below the symbol. Genotype GA/GA indicates that the NDM16 is carrier of W322* and G355R mutations, respectively. Clinical and genetic information for the uncoded family members is unavailable. (Family 3) In this family, two individuals are affected (filled symbols). The genotype for NDM17 for the *SCN4A* mutation is indicated below the symbol. Genotype GT indicates that the NDM17 is carrier of T1313M mutations. Clinical and genetic information for the uncoded family members is unavailable.

The study included three probands (NDM15-Family 1, NDM16-Family 2 and NDM17-Family 3, Figure 1) and 14 relatives of NDM15, all from three apparently unrelated families. Probands NDM15 and NDM16 were previously misdiagnosed with myotonic dystrophy type 1 (DM1). Most of the 17 individuals were clinically affected (EMG+), showing symptoms suggestive of a myotonic condition. Several members of family 1 were clinically re-evaluated and diagnosed with DMC, including NDM1, NDM2, NDM4-10 and NDM15 (Figure 1). Clinical data from these affected individuals have been described elsewhere [17] and a summary can be found in Supplementary Materials. The study was conducted in accordance with the Declaration of Helsinki, and signed informed consent was obtained for all subjects in accordance with the ethical protocols approved by the Ethical Committee of the University of Costa Rica (authorization VI-5449-2013).

### 2.2. Clinical Diagnosis

Clinical data were obtained after reviewing clinical records for proband NDM16 (Family 2; there is no information from EMG) and after reviewing clinical records, performing physical, motor nerve conduction and EMG tests (clinical re-evaluation) for patient NDM17 (Family 3). Data on motor nerve conduction test, including distal motor latency, motor nerve conduction velocities, F-M latencies, and amplitude of the compound muscle action potential (CMAP) of the median, ulnar, tibial, and peroneal nerves, were collected. We also performed a sensory nerve conduction test of the right median, ulnar and sural nerves. All tests were performed at skin temperature of 31 °C [13].

### 2.3. Genetic Analysis

Previously collected DNA samples from probands (NDM1, NDM15-17, Figure 1) were screened for mutations in *CLCN1* and *SCN4A* by Sanger sequencing. The encoding sequence of *CLCN1* (RefSeq NM_000083.3) and *SCN4A* (RefSeq NM_000334.4) were amplified by standard polymerase chain reaction (PCR). PCR conditions for *CLCN1* have been published elsewhere [18], and for *SCN4A*, they were optimized in our laboratory. Primers flanking the 24 *SCN4A* exons were designed using Primer3 and OLIGO 7 software and synthesized by Macrogen (Korea). Primer sequences and annealing temperature are provided in Supplementary Tables S1 and S2. PCR products were purified using the QIAquick PCR purification kit (QIAGEN, USA) and sequenced by Macrogen. Sequences were analyzed using the 4Peaks program (mekentosj.com) (Supplementary Materials Figure S1). Using restriction fragment length polymorphism assay, we confirmed/identified the genetic variants in probands, relatives or unaffected individuals. PCR products were digested with the respective enzyme, and genotypes were established by analyzing the band patterns in agarose gels stained with ethidium bromide. Finally, we used the multiplex ligation-dependent probe amplification (MLPA) array to identify duplications/deletions in the *CLCN1* and *SCN4A* genes. For details see Supplementary Materials.

### 2.4. Molecular Biology

The *CLCN1* mutations were introduced in human ClC-1 in the pTLN vector [12] using the QuikChange II XL Site-Directed Mutagenesis Kit following manufacturer’s instructions (Agilent, Folsom, CA, USA). For expression in *Xenopus laevis* oocytes, cRNA of ClC-1 was transcribed by the mMessagemMachine SP6 kit (Ambion—Life Technologies, Waltham, MA, USA) after linearization with MluI. The construct encoding the rat Na_v_1.4 channel and the beta1 subunit of the sodium channel, cloned in the pGEM oocyte expression vector, were kindly provided by Dr. Steve Cannon (Brain Research Institute, UCLA, Los Angeles, CA, USA). The *SCN4A* mutation was introduced by standard restriction-free mutagenesis. For expression in *Xenopus* oocytes, cRNA of *SCN4A* and beta1 plasmids were transcribed by the mMessagemMachine T7 kit after linearization with NheI. Constructs were verified by Sanger sequencing.

### 2.5. Heterologous Expression and Electrophysiological Recordings

The experiments were conducted under approval of the Institutional Animal Care and Use Committee of the University of Costa Rica (authorization CICUA-01-10) and the Italian Ministry of Health (authorization 638/2015-PR). For the experiments on oocytes in Genova, all animal protocols conformed to the European Community Guidelines on Animal Care and Experimentation and were approved by the Ethics Committee for Animal Experimentation of the Biophysics Institute. *Xenopus* oocytes were obtained from 2–5 years old frogs. Oocytes were injected/co-injected with ClC-1-cRNA, WT and mutant; or were co-injected with *SCN4A*-cRNA (WT or mutant) and beta1-cRNA and were incubated 48–72 h at 18 °C. Details on the solutions, protocols and equations can be found in Supplementary Materials. Briefly, once the oocytes expressed the ClC-1 or the Na_v_1.4 channels they were impaled by two KCl-filled electrodes. For measuring Cl^−^ and Na^+^ currents, we used the two-electrode voltage clamp technique at room temperature following previously published protocols [13,19,20]. ClC-1 currents were elicited at different voltage steps after a +60 mV prepulse, tail currents were fitted with a modified Boltzmann function, and the open probability (Po) was calculated following a standard approach. For Na_v_1.4 we determined the steady-state activation by fitting the peak currents obtained at different voltage steps, the steady-state inactivation through a –20 mV test pulse preceded several conditioning voltages steps (both fitted to modified Boltzmann functions), and the time course of recovery from inactivation after variable time periods at −90 mV, fitted to an exponential function. Recordings were performed with a TEC10CD amplifier (npi electronic GmbH, Tamm, Germany), using the custom acquisition program GePulse. Data analysis was performed using the custom analysis program Ana. Programs are available at http://users.ge.ibf.cnr.it/pusch/programs-mik.htm.

### 2.6. Structural Analysis of CLCN1 and SCN4A Gene Mutations

Structures of Na_v_1.4 (PDB ID: 6AGF) and ClC-1 (PDB ID: 6QV6) had been determined by Cryo-EM [21,22]. The Missense3D platform was used to predict the effect of the mutations at the structural level. DynaMut [23] was used to analyze the impact of mutations on protein dynamics and stability. WT and mutated structures were analyzed with PyMOL. We determined the clash score (defined as the number of serious steric overlaps (>0.4 Å) per 1000 atoms [24]) for some of the mutations.

Pore paths for WT and mutant proteins were analyzed in the MOLE*online* server [25]. The trajectories (distance and radius of the trajectory) and physicochemical properties (load, hydrophobicity, polarity, lipophilicity) were compared for each pore on the WT and mutant channel. For Na_v_1.4, the probe radius (10.0 Å) and interior threshold (1.0 Å) settings were used.

### 2.7. Statistical Analysis

Statistical analysis was performed using GraphPad Prism version 5 (San Diego, CA, USA). Normal distribution was assessed by Kolmogorov–Smirnov and Shapiro–Wilk normality tests and F-test was used to compare variances. Statistical significance between groups mean was assessed using Student’s *t*-test of two tails and analysis of variance (ANOVA, one-way or two-way where appropriate) followed by the Bonferroni post-hoc test. Data are expressed as mean ± s.e.m. Significance levels were set at 5%.

## 3. Results

### 3.1. Clinical Results

We performed a review of clinical records for proband NDM16 and a clinical re-evaluation for NDM17. Table 1 summarizes the detailed clinical picture found in these two probands (additional clinical information for NDM17 can be found in Supplementary Materials). We also included, in Table 1, clinical information of NDM15 and two affected relatives (NDM1 and NDM6, Figure 1), who were previously diagnosed with DMC [17]. However, some patients from family 1, such as NDM1 and NDM6, showed symptoms that have also been seen in SCM. Therefore, it is likely that SCM is also segregating in this family. After analyzing clinical data, the two new probands were diagnosed as: (1) RMC: proband NDM16 in Family 2; and (2) PC: proband NDM17 in family 3 (Figure 1, Table 1).

### 3.2. Molecular Results

No duplications/deletions were found in *CLCN1* or *SCN4A* (data not shown). Using Sanger sequencing of the two genes, the following results were obtained:

#### 3.2.1. Family 1

Proband NDM15 in family 1 (Figure 1) had been diagnosed with congenital DM1, since at birth he showed some DM1-like symptoms (Table 1). However, we could not find the CTG repeat expansion responsible for DM1 in this proband or in other key family members (NDM1, NDM2, NDM4 and NDM6). The analysis for detecting the DM1 mutation was carried out in a previous publication [26] by restriction-digested genomic blood DNA and Southern blot hybridization (using EcoR1 and BglI and the p5B1.4 gene probe or PstI and the probe pM10M6) following previous published methods [27,28,29]. All family members had shown a repeat size within the normal range (Supplementary Materials Table S3) [26]. Therefore, he was clinically re-evaluated and diagnosed with DMC with a severe MC phenotype. All family members clinically evaluated at that time (NDM1, NDM2, NDM4-10 and NDM15) were also affected with DMC (Figure 1) [17].

Screening of *CLCN1* led to the identification of a genetic variant (G-to-A base change) in exon 8, a nonsense mutation W322*, representing a novel *CLCN1* mutation. The proband turned out to be homozygous for this mutation (Family 1 Figure 1, Supplementary Materials Figure S1), with no other *CLCN1* mutation found. Regarding this variant, while no relevant information was found in several databases (National Center for Biotechnology Information (NCBI), Ensembl, ClinVar) at the time of publication, the variant was found in the dbSNP database (rs1441448091) and the gnomAD database with a reported frequency of 4 × 10^−6^. NDM15 is the result of a father-daughter incest, where both carrier parents transmitted the mutation. As mentioned before, proband NDM15 was much more severely affected than other family members, probably due to his homozygous status (Table 1). Because this genetic variant is likely to be the disease-causing mutation, we confirmed the variant in the genomic DNA of the proband. In addition, this genetic variant was absent in 100 unrelated, healthy or non-NDM affected individuals, showing a frequency of zero in the non-NDM population analyzed (Supplementary Materials Figure S2A).

DNA from additional 14 individuals (clinically re-evaluated or not) in this family was analyzed for this new mutation, which was found in eight family members in heterozygosity; three were clinically affected (NDM2, NDM5-6), but clinical information of the remaining family members (NDM3, NDM11-14) is not available (Family 1 Figure 1). The remaining six family members (NDM1, NDM4, NDM7-10), although clinically affected, resulted W322*-free (Family 1 Figure 1). Thus, we screened *SCN4A* in a second proband in this family (NDM1, Table 1, Figure 1). This led to the identification of a genetic variant (G-to-A base change) in exon 24, a missense mutation R1463H. The proband resulted heterozygous for this mutation (Family 1 Figure 1, Supplementary Materials Figure S1). Regarding this mutation, we found no relevant information in some databases (NCBI, Ensembl), but the gnomAD database reports a frequency of 8 × 10^−6^. Because this genetic variant could be the disease-causing mutation, we confirmed the variant in the genomic DNA of the proband. In addition, this variant was absent in 100 unrelated, healthy or non-NDM affected individuals, showing a frequency of zero in the non-NDM population analyzed (Supplementary Materials Figure S2B). All available DNA samples from this family were analyzed for this mutation. We found it in seven family members, all of them in heterozygous status (Family 1 Figure 1). Individuals NDM3 and NDM6 (both myotonic patients) carry both mutations (W322* and R1463H). However, at least for NDM6, the patient showed a similar phenotype (including age of onset, no weakness or muscle pain, generalized myotonia, etc.) as patients carrying only the *CLCN1* mutation in heterozygosity or the *SCN4A* mutation (see Table 1) [17]. In the case of NDM3 individual, who carry both mutations, she should be clinically affected, but clinical information for this individual is not available. Surprisingly, patient NDM10, even though clinically affected, did not carry either of the two mutations.

#### 3.2.2. Family 2

Proband NDM16 had been diagnosed with DM1, however, she carried a number of CTG repeats within the normal range (Supplementary Materials Table S3). A more careful review of clinically available data of proband NDM16 suggested that this patient was affected by NDM, compatible with RMC. Therefore, we screened *CLCN1* in this proband, which led to the identification of two different variants; the novel nonsense mutation W322* described in Family 1 (Figure 1), and an already reported missense mutation (G-to-A base change) located in exon 9, the G355R mutation (Supplementary Materials Figure S1) (with a reported frequency of 1.59 × 10^−5^ in the gnomAD database). The affected proband was heterozygous for both variants (Family 2, Figure 1), resulting in a compound heterozygous. According to the proband and her mother, no other affected family members (including the proband’s father) are present in this family, suggesting that the parents of NDM16 are asymptomatic. Clinical evaluation of the proband’s parents would confirm their clinical status. No additional relevant clinical information is available for this family at this moment, which together with more samples (none of them available at this moment), would allow the clinical phenotype and the genetic behavior of the mutation found in families one and two to be confirmed.

#### 3.2.3. Family 3

Proband from family 3 (NDM17) (Figure 1, Table 1) had been diagnosed with a myotonic condition (DMC or PC), but with little clinical information. To rule out a misdiagnosis, we determined the number of CTG repeats (in the *DMPK* gene), which was within the normal range (Supplementary Materials Table S3). Therefore, clinical re-evaluation and review of clinical records were performed. The clinical and exploratory picture of this patient is compatible with PC (Table 1, Supplementary Materials). By Sanger sequencing, we ruled out the possibility of carrying a mutation in *CLCN1*. However, a heterozygous G-to-C base change was found in exon 22 of *SCN4A* resulting in the known T1313M missense mutation (with a reported frequency of 4 × 10^−6^ in the gnomAD database) (Family 3 Figure 1, Supplementary Materials Figure S1). According to familial information, the proband’s father is affected, but no other known family member is affected.

### 3.3. Functional Characterization of Mutant Chloride Channels

**Figure 2:**
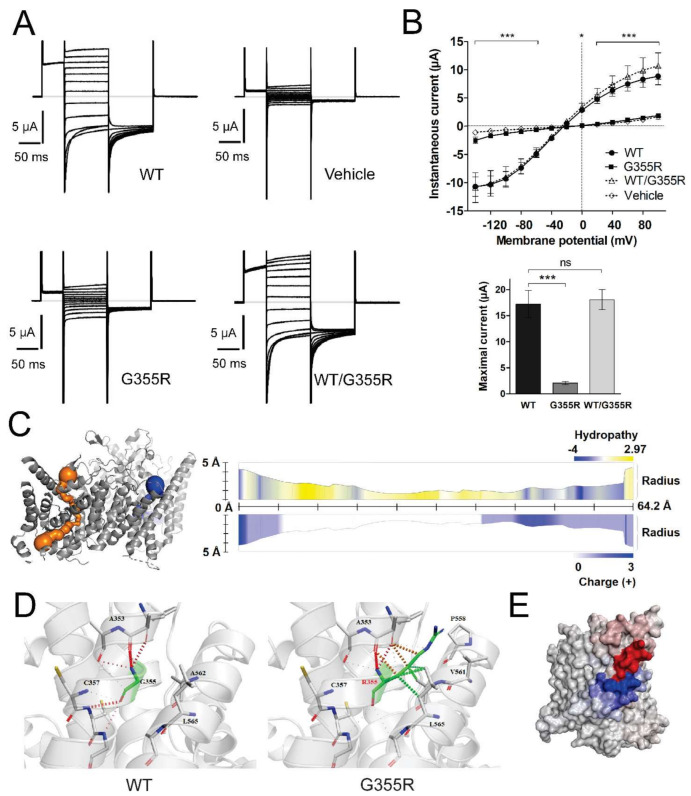
**G355R mutant channels characterization.** (**A**) Sample traces of macroscopic currents recorded in oocytes injected with the vehicle (water) or expressing wild-type (WT), homozygous (G355R) or heterozygous (WT/G355R) channels. (**B**) I-V relationship of the instantaneous current observed in the oocytes, the maximal currents from Boltzmann fitting are showed in the bottom panel. (**C**) Right panel shows the pore path prediction in MOLE*online* for ClC-1; left panel shows the dimeric channel reconstruction with both pores highlighted. (**D**) Amino acid interactions on WT (left panel) and predicted structural changes resulting from the G355R mutation (right panel); dotted lines represent amino acid bonds in or between alpha-helix structures. (**E**) Change of vibrational entropy upon mutation, where blue represents a rigidification of the structure and red a gain in flexibility. Values indicates mean ± s.e.m. * *p* < 0.05, *** *p* < 0.001 in G355R with respect to WT.

**Figure 3:**
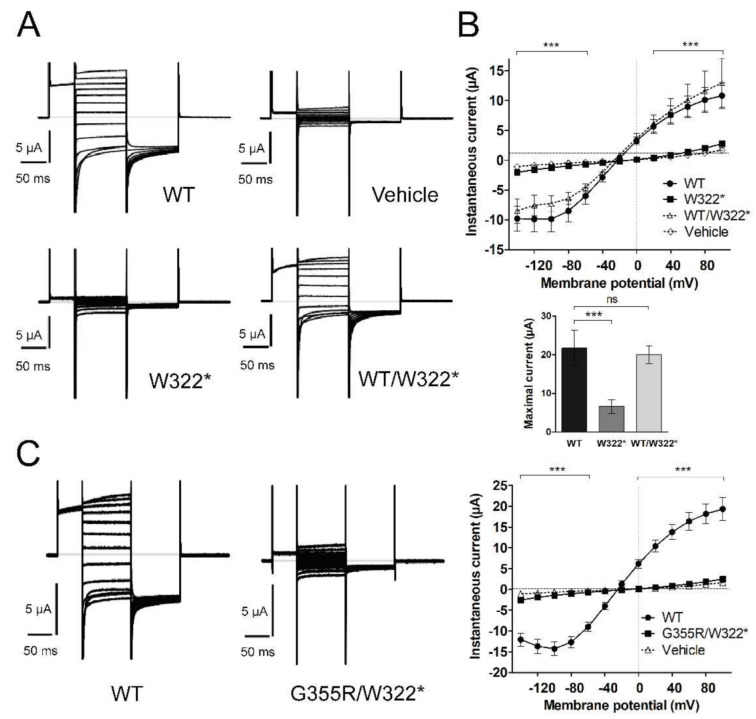
W322* mutant channels currents. (**A**) Sample traces of macroscopic currents recorded in oocytes injected with water or expressing WT, homozygous (W322*) or heterozygous (WT/W322*) channels. (**B**) I-V relationship of the instantaneous current observed in the oocytes, the maximal currents from Boltzmann fitting are showed in the bottom panel. (**C**) Sample recordings of the macroscopic currents recorded in oocytes injected with WT or with both mutants (G355R and W322*) in a 1:1 ratio and I-V relationship of the instantaneous current observed in these oocytes. Values indicates mean ± s.e.m. *** *p* < 0.001 in W322* or G355R/W322* with respect to WT.

WT ClC-1, and mutants G355R and W322*, were expressed individually or in different combinations to analyze their functional characteristics. When expressed alone, neither G355R nor W322* yielded functional macroscopic currents. The current-voltage (I-V) relationship showed that instantaneous G355R currents were smaller than those of WT and similar to currents observed in water-injected oocytes (mutation F_2,576_ = 4.81, *p* = 0.012; voltage F_12,576_ = 86.70, *p* < 0.0001; interaction F_24,576_ = 37.63, *p* < 0.0001 two-way ANOVA; *n* = 14 for WT, *n* = 16 for G355R and *n* = 21 for vehicle) (Figure 2A,B). Similarly, oocytes expressing W322* showed smaller currents than the WT channel, being similar to the currents observed in water-injected oocytes (mutation F_2,504_ = 0.015, *p* = 0.98; voltage F_12,504_ = 67.66, *p* < 0.0001; interaction F_24,504_ = 29.65, *p* < 0.0001 two-way ANOVA; *n* = 11 for WT, *n* = 13 for W322* and *n* = 21 for vehicle) (Figure 3A,B).

To test if these mutations behave recessively or dominantly, the same amount of WT and mutant cRNA were co-injected. Heterozygous WT/G355R was nearly superimposable with WT (heterozygous F_1,288_ = 4.29, *p* = 0.05; voltage F_12,288_ = 58.01, *p* < 0.0001; interaction F_12,288_ = 0.09, *p* = 1.00 two-way ANOVA; *n* = 14 for WT and *n* = 12 for WT/G355R) (Figure 2B). In the same way, heterozygous WT/W322* was statistically similar to WT (heterozygous F_1,264_ = 1.95, *p* = 0.18; voltage F_12,264_ = 38.47, *p* < 0.0001; interaction F_12,264_ = 0.08, *p* = 1.00 two-way ANOVA; *n* = 11 for WT and *n* = 13 for WT/W322*) (Figure 3B). In addition, comparing maximal instantaneous currents, both mutants reduced the current and both heterozygous were similar to WT (F_2,39_ = 26.7, *p* < 0.0001 and F_3,46_ = 14.1, *p* < 0.0001; respectively, one-way ANOVA) (Figure 2B and Figure 3B, bottom panel). Furthermore, we obtained the apparent open probability (Po) of WT and heterozygous WT/G355R and WT/W322*, and as expected from previous results, co-expression of each mutant with WT generated an activation profile like WT (Supplementary Materials Figure S3).

We also mimicked the compound heterozygous condition of proband NDM16 (Family 2) by co-expressing W322* and G355R in oocytes. Similar to the results obtained when each mutation was analyzed individually, the resulting macroscopic currents were smaller than WT currents, and similar to the currents in the water-injected oocytes (*n* = 16, *n* = 13, and *n* = 21 for WT, double mutant and water, respectively; mutation F_2,564_ = 4.51, *p* = 0.016; voltage F_12,564_ = 97.0, *p* < 0.0001; and interaction F_24,564_ = 62.00, *p* < 0.0001, two-way ANOVA) (Figure 3C). Also, the maximal instantaneous current was smaller than WT and similar to W322* (data not shown).

### 3.4. Functional Characterization of Mutant Sodium Channel

**Figure 4:**
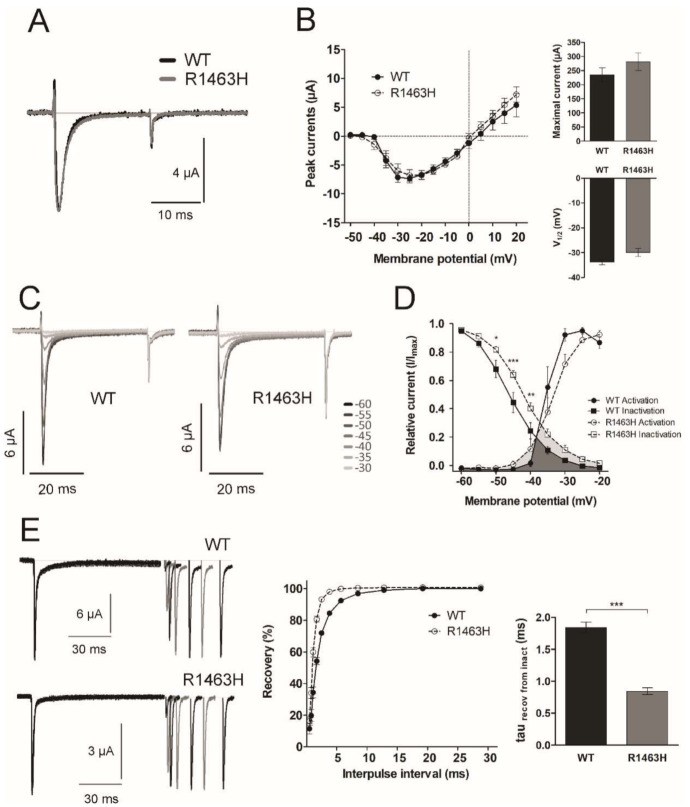
**R1463H mutant channels currents.** (**A**) Sample traces of macroscopic currents recorded in oocytes injected with the WT or with the mutant channel R1463H at −20 mV. (**B**) I-V relationship of the instantaneous current observed in the oocytes, maximal current and the voltage of half activation (V_1/2_) obtained by Boltzmann equation fitting are shown as inserts. (**C**) Sample traces at −20 mV for the steady-state inactivation after voltage steps from −60 to −30 mV. (**D**) Normalized I-V relationship for steady-state activation (circles) and inactivation (squares), the overlapping gray region of the curves represents the window current of the WT (dark gray) and R1463H (light gray). (**E**) Left panel shows sample traces of the currents developed during the protocol of recovery from inactivation in oocytes injected with the WT or with the R1463H channel, central panel shows the time course of the recovery of inactive channels, and the time constant (tau) of the recovery is shown in the right panel. Value indicates mean ± s.e.m. * *p* < 0.05, ** *p* < 0.01, *** *p* < 0.001 in R1463H with respect to WT.

We observed no evident changes in the current kinetics and I-V relationship between WT and mutant R1463H channels (Figure 4A,B) (*n* = 8 and *n* = 15 respectively; mutation F_1,294_ = 0.25, *p* = 0.63; voltage F_14,294_ = 45, *p* < 0.0001; and interaction F_14,294_ = 0.49, *p* = 0.94, two-way ANOVA). Also, by fitting the currents to Boltzmann equation, we found no statistical differences in the maximal current (t_21_ = 0.98, *p* = 0.34, *t*-test), the voltage of half-maximal activation (t_21_ = 1.67, *p* = 0.11, *t*-test) (Figure 4B, right inserts) or the relative current of activation (mutation F_1,126_ = 0.51, *p* = 0.48; voltage F_6,126_ = 109.1, *p* < 0.0001; and interaction F_6,126_ = 2.01, *p* = 0.07, two-way ANOVA) (circles in Figure 4D).

The inactivation kinetics (fast and slow tau) were also similar between the mutant and the WT channels at each voltage tested (data not shown). However, the steady-state inactivation of the R1463H channel was shifted to more positive voltages regarding WT (Figure 4C and squares in 4D) (*n* = 16, and *n* = 8 respectively, mutation F_1,176_ = 7.76, *p* = 0.01; voltage F_8,176_ = 450, *p* < 0.0001; and interaction F_8,176_ = 3.12, *p* = 0.003, two-way ANOVA). This result agrees with a more positive voltage of half-maximal inactivation from −45.7 ± 1.3 mV in WT to −41.5 ± 0.9 mV in the R1463H (t_22_ = 2.63, *p* = 0.02, *t*-test). This positive shift in the steady-state inactivation leads to 2-fold increase in window current in R1463H regarding WT channels (Figure 4D, lower overlapping gray regions). Functional analyses for the T1313M mutation were not performed due to this mutation having been widely characterized.

In addition, we tested the rate of recovery from inactivation, and mutant channels showed a faster recovery than WT channels (Figure 4E), with a time constant of recovery 50% smaller in R1463H (Figure 4E, right insert) (*n* = 10 for WT and *n* = 12 for R1463H, t_20_ = 10.0, *p* < 0.0001, *t*-test).

### 3.5. Structural Analysis of CLCN1 and SCN4A Gene Mutations

#### 3.5.1. Analysis of G355R Mutation

By using MOLE*online* analysis, we were able to predict and characterize the pore path of WT ClC-1 dimeric channel, but not for the mutant channel (Figure 2C). Despite this, Missense3D was able to determine that this amino acid substitution had a local clash score of 56.87, while for the WT was of 33.51, showing an increment in clash score >18 between the two channels. This can be derived from the replacement of a small buried uncharged residue (Gly, RSA 0.0%) with a bulky charged residue (Arg) with higher relative solvent accessibility (RSA 4.4%) (Figure 2D). In addition, the change in folding free energy (ΔΔG) was predicted destabilizing (−0.618 kcal/mol), and the change of vibrational entropy of this structure reveals an important reduction in molecule flexibility (ΔΔSVib: −1058 kcal ⋅mol^−1^ ⋅K^−1^) (Figure 2E).

#### 3.5.2. Analysis of R1463H and T1313M Mutations

**Figure 5:**
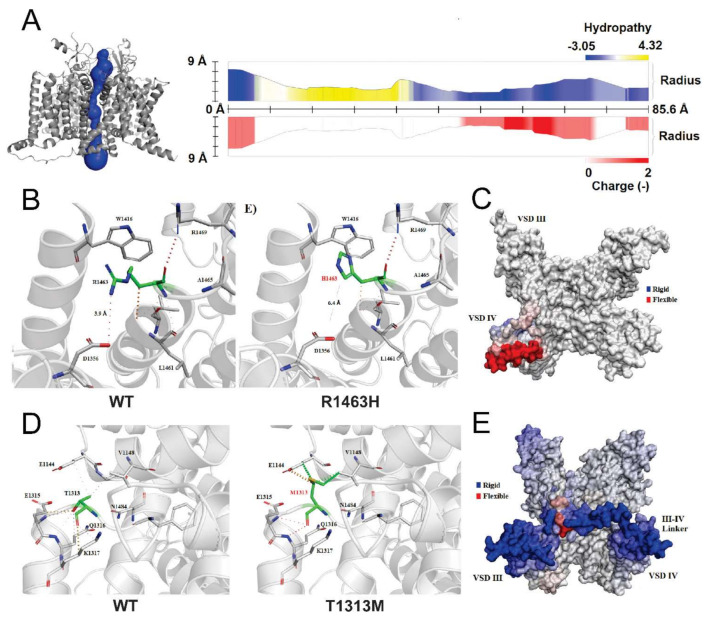
**Structural changes predicted for mutations R1463H and T1313M.** (**A**) Right panel shows the pore path prediction for Na_v_1.4 channel, the pore length was 85.6 Å with a radius <9 Å, and pore lining residues are negative as expected for a cation channel; left panel shows the channel reconstruction with pore highlighted. (**B**) Amino acid interactions on WT (left panel) and structural changes derived from R1463H mutation (right panel), where dotted lines represent amino acid bonds in or between structures. (**C**) Changes of vibrational entropy upon mutation, where blue represents a rigidification and red a gain in flexibility of the structure. (**D**) Amino acid interactions on WT (left panel) and structural changes predicted for T1313M (right panel). (**E**) Changes of vibrational entropy upon mutation, where blue represents a rigidification and red a gain in flexibility of the structure.

We used MOLE*online* server to predict and characterize the pore path of WT Na_v_1.4 (Figure 5A). Although Missense3D predicted that R1463H mutation leads to the shrinkage of volume cavity by 128,088 Å^3^, no significant changes in mutant pore path were determined. Moreover, we found that this mutation disrupted a salt bridge between the NH1 atom of R1463 and the OD1 atom of D1356 (distance: 3.9 Å) present in WT. In the mutant channel, the new distance between D1356 and the mutant H1463 is 6.4 Å, with no salt bridge (or any other type of bound) formed (Figure 5B). The change in folding free energy was predicted as destabilizing (ΔΔG: −0.400 kcal/mol) and the change of vibrational entropy energy between the WT and mutant structure is predicted to increase the flexibility of the molecule (ΔΔSVib: 0.150 kcal⋅mol^−1^⋅K^−1^), especially in the voltage sensor domain (VSD) IV (Figure 5C).

In the case of the T1313M, no structural characterization has been reported thus far. Therefore, to analyze the effect of this mutation in the channel structure, we also analyzed the pore path with MOLEo*nline* and structural insults with Missense3D. We did not find any structural damage for this mutation but noticed that the substitution involves the expansion of the cavity volume by 16.416 Å^3^. Like R1463H, the pore path analysis did not predict any change compared to the WT. However, this variant considerably affected amino acid interactions (Figure 5D) and decreased protein flexibility (ΔΔSVib: −0.328 kcal⋅mol^−1^⋅K^−1^), specifically in the III-IV linker (Figure 5E).

## 4. Discussion

It has been widely recognized that NDMs are not easy to distinguish, clinically or genetically, mainly due to the phenotypic overlap among these diseases and the great number of mutations with different effects, inheritance patterns and associated phenotypes [3]. In this study, we identified four genetic mutations in the *CLCN1* and *SCN4A* genes in three Costa Rican families and described their clinical and electrophysiological findings. In addition, little is known about the relationship between structural changes and the resulting functional effect in these channels. Hence, in this study, we integrated both approaches to achieve a better understanding of the behavior of the mutations found, to help distinguishing the NDMs.

The two *CLCN1* mutations identified (the new W322* and the already reported G355R [30]) are characterized by a complete loss-of-function. The lack of currents observed in the mutated ClC-1 channels will have profound effects on muscle cells excitability, leading to a MC phenotype, and supporting G355R and W322* as the disease-causing mutations.

Previously, it was reported that currents of the G355R mutated channel had a linear behavior independent of the chloride concentration, suggesting leakage [30]. As this substitution replaces a buried glycine, which is highly conserved in all mammalian ClC proteins, the channel stability seems significantly modified and the change is considered damaging [31]. Notably, the G > R change is classified as one of the most harmful variations [32] and likely to be disease-associated [33]. Unfavorable steric clashes and protein change in folding free energy are likely to cause the absence of currents. We speculate that the effect of the mutation on protein stability is so severe that the mutated subunit is unable to heteromerize with WT subunits, explaining the recessive nature of the mutation.

On the other hand, the W322* mutation produces a truncated ClC-1 protein, lacking about 2/3 of the sequence (reason why we did not perform structural analyses), likely leading to degradation before reaching the plasma membrane. In addition, it might be unable to heteromerize with WT subunits, explaining the recessive nature of this mutation [9]. It is likely that this degradation is achieved through nonsense-mediated mRNA decay [34]. The mRNA predicted for W322* contains a premature translation-termination codon (PTC) in exon 8, 14 bases upstream of 5′ intron 8. It has been shown that PTCs upstream an intron or inserting an intron downstream of a wild-type stop codon cause nonsense-mediated mRNA decay. In addition, more than 2000 bases remained between the new PTC and the end of *CLCN1* mRNA and long distances between the PTC and the poly(A) tail also induce mRNA decay. It has been reported that increasing the distance between a wild-type stop codon and the poly(A) tail causes a reduction in mRNA levels. Both mRNA characteristics trigger the recruitment of UPF1-3 proteins and other trans-acting factors that targets the mRNA to an accelerated destruction [34]. Yet, even if a transcript escapes mRNA degradation, it will be suppressed at the translational level because of abnormal secondary structures that can evoke low translation initiation rate, low translation elongation speed or high abortion rates [35]. These hypotheses can be tested for example by immunohistochemistry, or through protein or RNA quantification assays. However, the required samples to perform these analyses are not available at this moment. 

The co-expression of WT ClC-1 with any of these two mutations (mimicking the heterozygous condition) showed the recessive nature of the mutations, as functioning was unaltered. Therefore, heterozygous individuals for any of these mutations should remain asymptomatic. The recessive behavior for G355R had been described previously [36]. In this study, proband NDM16, who is compound heterozygous carrying these two mutations, was, in fact, diagnosed with RMC, indicating that neither allele can give rise to a functional ClC-1 channel. Similarly, proband NDM15, who resulted homozygous for the W322* mutation, should have been diagnosed with Becker’s type myotonia, but instead, he was diagnosed with severe DMC [17], indicating that the W322* mutation might be another example of *CLCN1* mutations associated with both phenotypes and inheritance patterns [3].

In the *SCN4A* gene, we identified two mutations segregating in families 1 and 3. T1313M is a well-known *SCN4A* mutation that has only been reported in patients affected with PC [37,38], including proband NDM17. The mutation affects a highly conserved part of the channel [39], increasing the activation at more negative voltages and impairing the fast inactivation of the channel, in a temperature-sensitive fashion [40]. Since this mutation was already functionally characterized, and it has been clearly associated with PC, we did not perform any functional analysis. However, we performed a structural analysis, which has never been done before. In this case, the mutation seems to trigger a strong decrease in molecule flexibility, as it is located in the III-IV linker, which is important for stability [41]. Interestingly, it has been demonstrated that deletion of the amino acids around T1313 (1307-GQDIFMTEEQ-1316) resulted in non-functional channels. This highlights the importance of this loop joining helices 48 and 49. Thus, as we confirmed for the first time in the structural analysis, it is highly likely that the decrease in flexibility of the III-IV linker caused by this mutation is the reason for the slower inactivation of the mutant T1313M Na_v_1.4 channel.

The R1463H mutation (found in family 1) was previously reported in a sporadic PC patient from The Netherlands [42], but no functional studies were performed, and no additional information was provided. By comparing the mutated with the WT channel, we observed a positive shift in the voltage-dependence of steady-state inactivation in R1463H channels, resulting in an increased window current, together with a faster kinetics of recovery from inactivation. Interestingly, the same amino acid was found to be mutated in a 3 month-old case of sudden infant death syndrome. Similar to the R1463H, the R1463S variant also showed a positive shift in the voltage-dependence of inactivation and a faster recovery from inactivation [43]. The possibility that the R1463H mutation might also increase the risk of sudden infant death needs to be investigated. Other Na_v_1.4 mutations linked to myotonic disorders have presented similar I-V patterns regarding WT channels, and faster recovery from inactivation [44]. A faster recovery at a physiological voltage has been shown for other mutations located near the S4-S5 linker of domain four [21,39,45], where the R1463H mutation is located.

R1463 is one of the six arginine “gating charge” residues responsible for voltage sensing in VSD IV [21]. It has been described that at resting potential, these residues are attracted to the cytoplasmic side; during depolarization the movement of this segment is particularly necessary for binding of the inactivation particle, and during repolarization, the movement of the VSD IV is thought to be the rate-limiting step for the recovery from inactivation [46]. This role has been specially proved for the first four arginine residues in the VSD IV (R1-R4) [46], but here, we show that R6 (corresponding to R1463) also has an important function in the channel flexibility. Therefore, it is likely that this mutation affects the outward movement of gating charges and the stability of the immobilized state of the VSD IV, with consequences in the stability of the inactivated state. It is known that factors that limit Na^+^ channel inactivation or induce more rapid reactivation following each spike, lead to a decrease in the accumulation of inactive channels and facilitate sustained high-frequency firing [47,48,49]. Model simulations have shown that modest gain-of-function changes in Na_v_1.4 are sufficient to produce myotonic responses [2]. This has also been shown for Na_v_1.9, where positive shifts in the steady-state inactivation of 4 mV are enough to significantly increase Na^+^ currents in ramp protocols [50]. Since the larger window current can increase a sustained Na^+^ current, and the faster recovery facilitates a repetitive firing, both can be related with the muscular stiffness seen in MC.

Since all *SCN4A* mutations linked to myotonia induce a gain-of-function of Na_v_1.4, carriers of the R1463H mutation should be diagnosed with sodium channelopathy, PC or SCM. Interestingly, we found this mutation in a family in which all affected members were diagnosed with DMC [17]. In this family, the affected members showed a very homogenous clinical phenotype, and all were almost equally affected (with the exception of the proband NDM15), including patients carrying only the dominant R1463H sodium mutation, the new recessive W322* chloride mutation or both. Due to clinical feature overlap between SCM and MC, it is still likely that in this family both diseases could be segregating. Another possibility is that R1463H could trigger both SCM and dominant MC phenotypes, being the first mutation reported thus far with this behavior and expanding the spectrum of mutations associated with the MC phenotype. The identification of more families with these mutations could contribute to clarify this issue.

Our functional data agrees with the phenotype observed in NDM16 (family 2, ClC-1 compound heterozygosity) and NDM15 (family 1, W322* homozygous). However, it suggests that heterozygous patients for W322* in family 1 should not be clinically affected. Although this mutation could be another example of *CLCN1* mutations with dual inheritance pattern [3], this is unlikely since the mutation results in an early truncated protein (and consequently non-functional ClC-1 protein). Since R1463H represents a gain-of-function, its presence can explain the myotonic condition in patients carrying this mutation alone (NDM1, NDM4 and NDM7-9) or in combination with W322* in heterozygous state (NDM3 and NDM6). Based on the genetic, clinical, and electrophysiological results, our data suggest that it is likely that another yet unidentified mutation is segregating in family 1, which could further explain the phenotype on patients who are heterozygous and carrying only the W322* mutation (NDM2 and NDM5), but also the phenotype of individual NDM10, who shows myotonic symptoms but does not carry neither the *SCN4A* nor the *CLCN1* variants identified in this family. Full exon sequencing of all affected individuals would be necessary to detect such an additional variant.

The coexistence of *CLCN1* and *SCN4A* mutations has been described previously in atypical sodium channel diseases [51,52,53], or as recently reported, in a patient affected by MC and a sodium channelopathy [54]. In the former cases, the *CLCN1* mutation might be acting as a disease modifier, and by triggering a synergic effect, could explain the atypical phenotype [3,51,52,53]. Therefore, it is likely that patients in family 1 carrying both mutations, a similar synergic effect could be observed, but in this case, the effect of both mutations on the function of the respective channel could trigger a Cl^−^ instead of a Na^+^ channelopathy. Another, relatively remote possibility is that particular *CLCN1* polymorphisms might modify the inheritance pattern and disease phenotype, especially by modifying ClC-1 function. To explore this possibility, we looked for possible SNPs in the available sequences of this family and identified two interesting missense variants (Supplementary Materials Table S4). These variants might modify the function of the channel and contribute to explain the inheritance pattern observed in this family. However, structural analyses of these two variants indicated that the amino acid change and more importantly, its location, does not seem to contribute to modifying the function of the channel (Supplementary Materials and Figure S4). Other two SNPs found in this family are sense variants relatively near splicing sites, which could contribute to modify the ratio of splicing isoforms. However, they would be expected to modify the phenotype instead of altering the inheritance pattern, while this family shows a homogeneous phenotype. Thus, also these two variants are unlikely to contribute to the disease.

Importantly, these families have not been tested for the mutation responsible for DM2, thus, we cannot exclude this possibility, at least in some family members. However, the clinical signs and history suggest that they are more likely affected by a non-dystrophic than a dystrophic myotonia. Future studies, such as exome sequencing, need to be carried to identify other putative mutations present in patients of family 1 and their functional implications on muscle excitability.

Finally, our data suggest that it must be stressed that genetic testing in myotonia patients should include both *CLCN1* and *SCN4A* genes, even if a mutation has been found in one of them.

## Figures and Tables

**Table 1:** Clinical features of five patients with *CLCN1* and *SCN4A* mutations.

Patient/Code	Sex	Onset	MuscularStiffness	MuscularPhenotype	EMG Test ^1^	Other Features	Most likely Clinical Diagnosis
F1-NDM1 ^a^	F	Youthhood	Yes, with warm-upphenomenon	- No muscle pain - No muscle weakness	- Positive for generalized myotonia (more evident in arms than in hands) - Typical myotonic discharges - Myotonia more evident in the proximal muscles than in the distal muscles	- No walking problems - Normal sensitivity	Thomsen’s disease
F1-NDM6 ^a^	F	Youthhood	Yes, with warm-upphenomenon	- No muscle pain - No muscle weakness - Hypertrophy of calves	- Positive for generalized myotonia (more evident in arms than in hands) and percussion myotonia - Typical myotonic discharges - Myotonia more evident in the proximal muscles than in the distal muscles - Ocular myotonia	- No walking problems - Adiadochokinesia - Difficulty to manipulate objects - Normal sensitivity	Thomsen’s disease
F1-NDM15 ^a^	M	Birth	- Yes, with warm-up - phenomenon - Hypotonia at birth	- No muscle pain - Weakness in the scapular waist muscles and in the neck flexors. - Hypertrophy of calves	- Positive for generalized myotonia (more evident in arms than in hands) and percussion myotonia - Typical myotonic discharges - Myotonia more evident in the proximal muscles than in the distal muscles - Ocular myotonia	- Walking problems - Motor problems after remaining at rest for a long time, however, as he exercised, these problems were disappearing little by little - Adiadochokinesia - Difficulty to manipulate objects - Frequent falls - Sign of Gowers, however, improved with the repetitions of the movement - Very poor myotatic reflexes - Normal sensitivity	Severe Thomsen’s disease
F2-NDM16	F	Childhood	Unknown	Muscle weakness	- Myopathic - Myotonic discharges associated with abundant denervation activity	- Difficulty standing, and climbing stairs - Muscle weakness to get up when sitting for long time - No difficulty to manipulate objects - Can run and jump, but after exercising feel very tired	Becker’s type myotonia
F3-NDM17	M	Youthhood	- Yes without - warm-up - phenomenon - No muscle amyotrophy	- With muscle pain (arms) - Muscle weakness - Biotype similar to Thomsen	- Positive for myotonia - (orbicularis oculi muscle and hands) - Myotonic phenomenon increases because of continuing exercise - Long myotonic and pseudomyotonic discharges - Percussion myotonia in right deltoid	- Symmetric poor myotatic reflexes - Touch hypoesthesia: glove and stocking distribution - Insomnia, fragmentation of sleep (sleep apnea?) - Centripetal obesity - Normal sensitivity - - Normal gait	Paramyotonia congenita

*CLCN1* gene, RefSeqNM_000083.3, SCN4A gene, RefSeqNM_000334.4. ^1^ EMG, electromyography. ^a^ Clinical data taken from a previous published work by our research team [13].

## Data Availability

The data presented in this study are available in the figures and tables of the manuscript and supplementary materials available online at www.mdpi.com/xxx/s1.

## Supplementary Materials

The following are available online at https://www.mdpi.com/2073-4409/10/2/374/s1, Supplementary Materials and Methods: Clinical picture of Family 1, RFLP and MLPA assays, Heterologous expression and electrophysiological recordings, Additional clinical information for subject NDM17, Polymorphisms NP_000074.3:p.Gly118Trp and NP_000074.3:p.Pro727Leu. Table S1: *SCN4A* primers and PCR conditions used in this study, Table S2: *CLCN1* primers and PCR conditions used in this study, which were reported previously, Table S3: Sizes of the CTG repeat expansion obtained by Southern blot hybridization or PCR for the tested patients, Table S4: Single nucleotide polymorphisms (SNP) found in Family 1, Figure S1: Sanger sequencing showing the mutations found, Figure S2: Genotyping assay for the two new mutations found. A. Genotyping of the *CLCN1* gene mutation, W322*, Figure S3: Open probability (Po) of the heterozygous oocytes, Figure S4: Structural variations derived from G118W and P727L variants in the ClC-1 channel.
