# Supplementary material for: Functional and Structural Characterization of ClC-1 and Nav1.4 Channels Resulting from CLCN1 and SCN4A Mutations Identified Alone and Coexisting in Myotonic Patients
Source: Cells. 2021 Feb 11;10(2):374. doi: 10.3390/cells10020374 (PMC7918176; doi:10.3390/cells10020374)
Supplement: Supplementary file 1 [file cells-10-00374-s001.pdf]

Supplementary information for:

Functional and structural characterization of ClC-1 and $Na_V1.4$ channels resulting from *CLCN1* and *SCN4A* mutations identified alone and coexisting in myotonic patients

Oscar Brenes [1,2], Raffaella Barbieri [3], Melissa Vásquez [4], Rebeca Vindas-Smith [4], Jeffrey Roig [4], Adarli Romero [5], Gerardo del Valle [6], Luis Bermúdez-Guzmán [7], Sara Bertelli [3,8], Michael Pusch [3,*], Fernando Morales [4,*]

1 Departamento de Fisiología, Escuela de Medicina, Universidad de Costa Rica; oscar.brenes_g@ucr.ac.cr
2 Centro de Investigación en Neurociencias (CIN), Universidad de Costa Rica
3 Istituto di Biofisica, CNR, Genova, Italy; raffaella.barbieri@ge.ibf.cnr.it; sara.bertelli89@gmail.com; michael.pusch@ibf.cnr.it
4 Instituto de Investigaciones en Salud (INISA), Universidad de Costa Rica; melissa.vasquez@ucr.ac.cr; rebeca.vindas@ucr.ac.cr; jsroigf@yahoo.com; fernando.moralesmontero@ucr.ac.cr
5 Escuela de Biología, Universidad de Costa Rica; adarli.romero@ucr.ac.cr
6 Laboratorio de Neurofisiología (Neurolab); neurolab22@yahoo.com
7 Sección de Genética y Biotecnología, Escuela de Biología, Universidad de Costa Rica; luis.enriqbg@gmail.com
8 Scuola Internazionale Superiore di Studi Avanzati (SISSA), Trieste, Italy
* Correspondence: fernando.moralesmontero@ucr.ac.cr; Tel.: +506-2511-2124 (F.M.); michael.pusch@ibf.cnr.it; Tel: +39-0106475-553/522 (M.P.)

1. Supplementary Materials and Methods

1.1. Clinical picture of Family 1 (Figure 1) [1]

Since this family was presumptively affected with myotonic dystrophy type 1, we studied several features typical for DM1, such as cataracts. The ophthalmological examination was performed on four individuals, NDM1, 2, 6 and 15, who resulted cataract-free and none reported visual disturbances. Therefore, we did not perform this study in the remaining relatives.

There was no family history related to cardiac problems. Nevertheless, we performed electrocardiograms in 10 individuals (NDM1, 2, 4, 5, 6, 7, 8, 9, 10, 15), who showed a normal pattern.

None of the family members evaluated reported muscle pain. Only the proband (NDM15) had walking problems characterized by frequent falls and resulted to be the most affected individual in the family. NMD15 presented motor problems after resting for a long time. However, when he made repeated movements, these problems gradually disappeared (warm up phenomenon). Individuals NDM6 and NDM15, showed adiadochokinesia. NDM6 and NDM8 showed difficulties to manipulate objects with their hands. Individual NDM5 had muscle cramps.

None of the family members reported digestive issues or sleep disturbances.

Almost all individuals showed normal muscle strength. The proband (NDM15) showed weakness in the scapular waist muscles and in the neck flexors.

All the individuals were positive for generalized and percussion myotonia. Myotonia was more evident in arms than in hands, and in the proximal than in the distal muscles. Individuals NDM6 and NDM15 also showed ocular myotonia and hypertrophy of calves. The proband NDM15 also presented the Gower's sign. Very poor myotatic reflexes were found in individuals NDM8 and NDM15, and fragmented eye movements were also found in patient NDM8. All individuals had normal sensitivity. Due to lumbar radiculopathy, patient NDM2 showed reflex asymmetry in the lower limbs.

Thus, clinical signs of all evaluated patients were compatible with a myotonic disease, more specifically to dominant myotonia congenita.

1.2. RFLP and MLPA assays

By using the restriction fragment length polymorphism (RFLP) assay, we were able to confirm/identify the genetic variants in the probands, relatives or NDM unaffected individuals. The *CLCN1* mutation, NM_000083.3:c.1063G>A, NP_000074.3:p.(G355R), eliminates an AvaI restriction site (data not shown); the *SCN4A* mutation, NM_000334.4:c.3938C>T, NP_00325.4:p.(T1313M) creates a BccI restriction site (data not shown); for the new *CLCN1* mutation, NM_000083.3:c.966G>A, NP_000074.3:p.(W322*), by using a restriction-specific primer on a nested PCR, we created an AvaII restriction site (Figure S2A); the *SCN4A* mutation, NM_000334.4:c.4388G>A, NP_00325.4:p.(R1463H), eliminates a SacII restriction site (Figure. S2B). For the new unreported and functionally undescribed variants, we screened 100 healthy unrelated individuals or with a disease different to NDM.

The multiplex ligation-dependent probe amplification (MLPA) assay was performed on all 17 samples involved in this study. For this, we used the MRC-Holland kits: 1- SALSA MLPA 350 CLCN-1 - KCNJ2 for *CLCN1* gene; and 2- SALSA MLPA P397 *SCN4A* - CACNA15 for *SCN4A* gene, following the manufacturer's instructions. Approximately 30ng of DNA was used to perform the MLPA assay. To perform the hybridization of the probes, DNA was incubated for 5 min at 98 °C and subsequently the probe mix and the MLPA buffer were added. The reaction was incubated for 1 min at 95 °C and then kept at 60 °C for 18 hours. For the ligation, the enzyme ligase, the ligase buffers A and B and the DNA (from previous step) were used. The reaction was incubated at 54 °C for 15 min, 98 °C for 5 minutes and then kept at 20 °C. For the amplification of the probes, 8 μl of the previous reaction, water, the primers and the

DNA polymerase were added and amplified in the thermal cycler with the following conditions: 95 °C for 30 s, 60 °C for 30 s and 72 °C for one minute (x33), 72 °C for 20 minutes, and then kept 15 °C. MLPA products were sent to the Macrogen (Korea) for sequencing. The analyzes of the results were carried out with the Coffalyser program of MRC Holland.

1.3. Heterologous expression and electrophysiological recordings

Xenopus oocytes were obtained from 2-5 years old frogs and defolliculated by digestion with collagenase from Clostridium histolyticum (Sigma-Aldrich, USA). Oocytes were injected/co-injected with ClC-1-cRNA (WT/mutant) or were co-injected with *SCN4A*-cRNA (WT or mutant) and beta1-cRNA and incubated 48-72 h at 18 ºC in a maintenance solution containing (in mM) 90 NaCl, 2 KCl, 1 $MgCl_2$, 1 $CaCl_2$, and 10 Hepes (pH 7.5). In order to measure chloride currents, the membrane potential was kept at the holding potential of -30 mV and subjected to voltages stimuli consisting of a prepulse to +60 mV for 50 ms followed by different voltage steps from -140 to +100 mV in 20 mV increment for 100 ms, and a final tail pulse to -100 mV for 100 ms. The composition of the extracellular solution (in mM) was: 100 NaCl, 10 HEPES-Na, 5 $MgSO_4$ (pH 7.3) and the electrodes were filled with 3 M KCl.

The initial tail currents obtained by back-extrapolating single exponential functions fitted after the decay of the capacitive current to the onset of the voltage step were fitted with a modified Boltzmann function of the form:

$$I(V) = I_{max}(p_{min} + \frac{1-p_{min}}{1+exp^{(V_{\frac{1}{2}}-V)/k)}}) \qquad (1)$$

where I_{max} is the maximal current extrapolated by the fitting, p_{min} is the residual open probability at negative voltages, $V_{1/2}$ is the voltage of half-maximal activation, and k the slope factor of the voltage dependence of the open probability. The apparent open probability (Po) was calculated by the normalization:

$$Po = \frac{I(V)}{I_{max}} \qquad (2)$$

where I(V) are the currents fitted at each voltage step.

Sodium currents were measured with different protocols and the extracellular solution had a reduced sodium concentration to reduce the magnitude of inward currents and the reliability of the voltage-clamp (in mM): 15 NaCl, 100 NMDG-Cl, 10 Hepes, 1.8 $CaCl_2$, 1 $MgCl_2$ (pH 7.3).

The membrane was kept at a holding potential of -90 mV. The standard I-V protocol was performed by applying 20 ms long pulses at voltages ranging from -50 to +20 in 5 mV increment. The steady-state activation was determined by fitting the peak current-voltage (I-V) relationship with the equation:

$$I(V) = \frac{g_{max}(V-V_{rev})}{1+exp^{-z_{act}(V-V_{1/2})F/RT}} \qquad (3)$$

where V_{rev} is the reversal potential, g_{max} the maximum conductance, z_{act} the apparent gating valence, F the Faraday constant, R the gas constant and T the temperature. The parameter g_{max} is a measure of the functional expression level.

Steady-state inactivation (channel availability) was measured after 100-ms conditioning pulses to various voltages followed by a test pulse to –20 mV for 40 ms, and currents were fitted with the equation:

$$I(V) = \frac{I_{max}}{1+exp^{z_{inact}(V-V_{1/2})F/RT}} \qquad (4)$$

where V represents the pre-pulse potential, I_{max} is the maximal current, $V_{1/2}$ the voltage of half-maximal inactivation, and z_{inact} is apparent valence of the inactivation gate.

The voltage-dependent activation and the steady state inactivation were plotted as a function of voltage and the area subtended by the intersection of the curves was calculated and defined as window current.

The time course of recovery from inactivation at -90 mV was measured by repolarizing the cell to -90 mV for a variable time from 0.5 to 100 ms after a 100 ms pulse to 0 mV and assessing channel availability

by a final test-pulse to -10 mV. Peak-currents at the final test pulse were analyzed by fitting an exponential function:

$$I(t) = I_{max} - I_r e^{-\frac{t}{\tau_r}} \qquad (5)$$

Where τ_r is time constant of recovery. Linear capacitive and leak currents were online subtracted using a standard P/4 protocol.

1.4. Additional clinical information for subject NDM17

NDM17 reported progressive muscle weakness and stiffness in his hands, showed myotonia, which got worse with exercise. The motor nerve conduction study resulted normal, with mild decreases in the CMAP of the right ulnar, while the sensory nerve conduction study showed a slight decrease in the amplitude of the right ulnar potential and a severe decrease in the amplitude of the right sural potential.

1.5. Polymorphisms NP_000074.3:p.Gly118Trp and NP_000074.3:p.Pro727Leu

Structural analysis of these two genetic variants indicated that:

1- G118W had a low disease-propensity score (40/100) and missense3D did not predict any structural damage. In addition, the ΔΔG is predicted to be stabilizing (0.526 kcal/mol). Although it might seem that the impact of changing a glycine to a tryptophan can be severe, there is a neighboring helix that also has a tryptophan (W303) in a similar spatial location, suggesting that there is a kind of structural accommodation. This variant is only reported once in ClinVar and it is classified as "benign".

2- P727L had a very low disease-propensity score (11/100) and missense3D did not predict any structural damage. Although predicted stabilizing (ΔΔG: 0.399 kcal/mol), the mutation is located downstream of the first cystathionine β synthase (CBS) domain, in a disordered region. This variant is also classified as "benign" in ClinVar. Interestingly, proline at position 727 is highly conserved (in a random coil), therefore, a change in this position could generate an important modification in the channel's function. However, the structural analysis performed does not suggest that.

3- Accordingly to these results, it is not expected that these variants can severely affect the structure and function of the ClC-1 channel.

2. Supplementary Tables and Figures

Table S1. *SCN4A* primers and PCR conditions used in this study.

NAME	FORWARD PRIMER	REVERSE PRIMER	AT	FS
SCN4A-1A	5′-AGCAGGAGGTGAGGAGTGG-3′	5′-GTCACTTCGTGGCTTCCGTT-3′	55.5	294
SCN4A-1B	5′-TCACTGGCAGCCATAGAACA-3′	5′-TGGATGGCAGACAGACAGAG-3′	55.5	245
SCN4A-2	5′-CATCCATCTGTCTGCCTGT-3′	5'-CATCTCGCCCATCCCTAAC-3′	59.3	229
SCN4A -3	5'-ACCTCCCATCCTAGCTGCTT-3′	5′-TCAGGGAGCAGGGAGACTT-3′	60	157
SCN4A-4	5′GTTGCCACACTGACCCCTAC-3′	5'-ACCCCAGCCTCAGGATGTC-3′	59.3	226
SCN4A-5	5'-CCCAATTTCTTGGGAATCCT-3′	5'-TCTCTCAGCTCAGGCAGAGG-3′	51.1	240
SCN4A-6A	5'-GCCAATATCCTTGCCCTCTC-3′	5'-CGTTGGCATACCATGAGT-3′	59.3	290
SCN4A-6B	5'-TTCAACGACACCAACACCAC-3′	5'-CGTCACCCTCCCCATTCTT-3′	55.5	199
SCN4A-7	5'-TGTGTCCATGTGGGTGACTT-3′	5'-TAATCAGCTCCCACCTTCCA-3′	59.3	176
SCN4A-8	5′AGCCCCCTGTCCTATTCCTA-3′	5'-TGAAGAGGCTGCAGGGATG-3′	59.3	230
SCN4A-9	5'-CACTCCCTCTCCCTCCACTC-3′	5′CCCTGGGTCCTCTATCTCCT-3′	59.3	270
SCN4A-10	5'-CTCTGGCTCAGAGCTGGACT-3′	5'-AGGCTCCACCCCTACCTAAG-3′	59.3	249
SCN4A-11	5′ACCTGCTACCACCCCTCTC-3′	5'-CCTGCCCATGAATGATCC-3′	62.1	300
SCN4A-12	5'-CTCTGTGACAGGGCCTCAT-3′	5'-CCCTGGGGCTTTTGTGTA-3′	62.1	224
SCN4A-13A	5'-CTGGGCCTCATTCACCCTT-3′	5'-GACGATGAGGAAGGAGTGGA-3′	59.3	280
SCN4A-13B	5'-AGCTACAAGGAGTGCGTGTG-3′	5'-GAGAGGATGTGTTGGGGA-3′	52.9	302
SCN4A-14A	5'-TGTGCTGGCTGAAGGTGG-3′	5'-CAGCAGCCCCAGGAGGAA-3′	64	228
SCN4A-14B	5'-GATGAGGATGGCGAGATG-3′	5'-CCCATGTGGTTCAGGATGT-3′	51.1	254
SCN4A-14C2	5'-GGAGGACCTGAAGAAGGACA-3′	5'-GAGAATTTGAAGCCGGGTCT-3′	55	254
SCN4A-15	5'-GATGTCATGTGACCCTGTGG-3′	5'-GCTTTTGGTCCTGGTGTAGC-3′	53	248
SCN4A-16	5'-CATGATGTGGTGCCTGTCC-3′	5'-CCCTTCCTGTGTGTGGAGAC-3′	59.3	286
SCN4A-17	5'-GAACCAGCAGCATAGAGCAG-3′	5'-TGACAGCCTCTGGATGTAGC-3′	65.0	308
SCN4A-18	5'-GCTGTGAGTCCAGGAAGA-3′	5'-GAGTATAGACATGCACCCTCCA-3′	59.3	298
SCN4A-19A	5'-GTGGCGTAGAGATGTGGAG-3′	5'-TGTTGTTGACCTCGGAGATG-3′	59.3	247
SCN4A-19B	5'-CATCAACACCACCACCTCT-3′	5'-TGACACTGGGGTTGGGTA-3′	59.3	240
SCN4A-20	5'-CTCCCCAACCACACTCTCTC-3′	5'-GCCAAGTCTCCCTCCTGTCT-3′	62.1	150
SCN4A-21	5'-AGAAGCCAGCGTGCAAACC-3′	5'-ACAAAGGAGGCAGGAGGGAG-3′	63.9	248
SCN4A-22	5'-GGGAACTGCACCTCAACCT-3′	5'-ATCAGTCCCCTAGGCAGGAG-3′	65.0	207
SCN4A-23A	5'-CCAACTCCTCCACATCCACT-3′	5'-ACAGGATGTCCACCTTGAGC-3′	59.3	175
SCN4A-23B	5'-CCAGCTCAAGGTGGACAT-3′	5'-AGGATGGGTCTTCCCGAGT-3′	59.0	219
SCN4A-24A	5'-GCAGCGTCCTCACTAGCTTC-3′	5'-ATGCCCGACTCCTTCTTGAC-3′	59.3	279
SCN4A-24B	5'-TCCTCCTCTTCCTGGTCA-3′	5'-AAGCAGATGCCGATGGAG-3′	55.5	271
SCN4A-24C	5'-CCCAGACTGTGACCCCAA-3′	5'-TGGCACCATGGGCAAGTC-3′	59.3	361
SCN4A-24D	5'-GATTGCCAAGCCCAACAAGA-3′	5'-CGGTAGGCCCTCTGGATCTT-3′	59.3	261
SCN4A-24E	5'-GTGTCCTACGAGCCCATCAC-3′	5'-GGCATCAGCCCCATAGTG-3′	63.0	261
SCN4A-24F	5'-CGAGAATGGGAACAGCAG-3′	5'-AGTGTGGCCAAATCCTGT-3′	52.9	308
SCN4A-24G	5'-TGAGGGAGGAGGGCTTTG-3′	5'-GGCAACTGATCCCTCCAC-3′	59.3	302
SCN4A-24H	5'-GAATCTCTGCCCCTCACT-3′	5'-GGTTTTGAGGCTTTGAGG-3′	55.5	295
SCN4A-24I	5'-CCAGGCCAGCTCAGGAAT-3′	5'-AGGCCATGTGCAGCGATC-3′	55.5	285
SCN4A-24J	5'-TTTTCTAGGAAGCACGGGGG-3′	5'-CCCCAGGGTCGAAATATCTA-3′	55.5	309
SCN4A-24K	5'-ACCTTTCCCACCTCTCTC-3′	5'-CACAGCTATACACACAGG-3′	55.0	306
SCN4A-24L	5'-GGCTGTGTGTCTGCTTGT-3′	5'-CACCTTACCACATGCGTT-3′	51.0	295

SCN4A-24M	5'-TGTGCTGTGAGTGTGTGG-3′	5'-AGAGGGGAGTGAGAGAGA-3′	55.0	298
SCN4A-24N	5'-ATGCTCTCTCACCTGCTC-3′	5'-AATGTGCCAGGAAGGGGAGA-3′	65.0	212
SCN4A-24O	5'-TTTCGTCTAATGGGGGCAGT-3′	5′-CATACCAGAGGCACCAAGGA-3′	55.0	309

AT: annealing temperature; FS: fragment size.

Table S2. *CLCN1* primers and PCR conditions used in this study, which were reported previously [2].

NAME	FORWARD PRIMER	REVERSE PRIMER	AT	FS
CLCN1-1	5′-GGGGCTCGGGGGGAGGGAAT-3′	5′-CCTCATTTTCACCAGTCTCT-3′	56	272
CLCN1-2	5′-CTTCCACAAGGCAGACACTG-3′	5′-ATGCCCAAGTTATTCTCCTA-3′	51	234
CLCN1-3	5′-TTTTCCCTCATCTCTTCCTA-3′	5′-CCATAACACACCCTGCTTAC-3′	53	256
CLCN1-4	5′-CGGTGGACACGGCTGCTCAG-3′	5′-GCCGAGTCTGGTGGCAAGTT-3′	59	211
CLCN1-5	5′-TAATCTTTCAACGCTTTTAG-3′	5′-ATCCACTTCCACTCCCAGAG-3′	53	248
CLCN1-6	5′-CCTCTGTGTAACTCCCGTAT-3′	5′-ATTTCACTGTCTCAACCTTA-3′	53	227
CLCN1-7	5′-ACCCACCCTGTTTCTCTGTC-3′	5′-GCTATTCTCGTAAGTAACCT-3′	51	227
CLCN1-8	5′-TGCCCCCAACCACACTTCTG-3′	5′-GCCCATTATTCCTTTTCTGA-3′	55	259
CLCN1-9	5′-ACTGGCCTTTCCATCCTACA-3′	5′-GCCTCACTCCACACCCTGCT-3′	54	157
CLCN1-10	5′-TCCAAGAGATGAGGATTTCA-3′	5′-GACAAAAAGGGAGGAACTCT-3′	52.5	229
CLCN1-11	5′-ATTTACTGTGAGTTGGCTGA-3′	5′-GTGCTGTTTCCTCTTTACCA-3′	52	178
CLCN1-12	5′-GACCACCTTCTGCTTCTTCC-3′	5′-TGGAGGTTTAGGGTGTTGCT-3′	54.5	214
CLCN1-13	5′-CAGAGTTGAAAGGGTATTCC-3′	5′-CCTTATGTTTCCTGTATCCT-3′	51	176
CLCN1-14	5′-ATCTCGTAACACCTTCCTTC-3′	5′-ATGGGAGAGTTTAAGTGTGG-3′	51	221
CLCN1-15	5′-CAGGCGTATTCCTGTGTCAT-3′	5′-ATTCCGTCTAGTGCCCCTGA-3′	56	260
CLCN1-16	5′-GGCTAACCCACCATGCTTCT-3′	5′-ATTCAGGTCACTAGCTCTGG-3′	55	209
CLCN1-17	5′-GCCTCTCCTGTTCCTTCTCA-3′	5′-AGACGACOCTTTCCCTTGCT-3′	59	389
CLCN1-18	5′-CCAGGCTGAGACTTCTTACT-3′	5′-AGGGGTGAGTTGGGGTGCAT-3′	55	179
CLCN1-19	5′-CATCCACTCACCTGTCCTCT-3′	5′-GGGTCTCTGCCTTCCTGATT-3′	54.5	197
CLCN1-20	5′-GGAAGAAAAGGGAAAGAACT-3′	5′-GACAACACAATAAAGAAGGT-3′	47	171
CLCN1-21	5′-TGATTTTCGTGACTTTCCTC-3′	5′-CCCCACAGCCCTGAGCAGTC-3′	54	172
CLCN1-22	5′-ACCTGTGCTCTTCATCCTCA-3′	5′-CCCTCCTGCTGCTCAAATGG-3′	55	144
CLCN1-23A	5′-TTTCCAACTTTTTACCCTCT-3′	5′-TGGGGAGGCAGCAATCACAT-3′	55	229
CLCN1-23B	5′-ACTGGAACAGGGGATGTGAT-3′	5′-TTTATGAGGAGGTCGTGGGA-3′	58	244

AT: annealing temperature; FS: fragment size.

Table S3. Sizes of the CTG repeat expansion obtained by Southern blot hybridization or PCR for the tested patients.

Patient	Restriction enzyme / Technique	Fragment size (kb) / CTG repeats
NDM1	BglI	3.4
	EcoR1	8.6
NDM2	BglI	3.4
	EcoR1	8.6
NDM4	PstI	1.2
	EcoR1	8.6
NDM6	BglI	3.4
	EcoR1	8.6
NDM15	BglI	3.4
	EcoR1	8.6
NDM16	PCR	12
		12
NDM17	PCR	5
		10

Table S4. Single nucleotide polymorphisms (SNP) found in Family 1.

Patient(s)	SNP	Variant	Exon	Patient´s genotype	Reference allele (frequency)	Putative effect
NDM1<br>NDM15	Rs6962852	Synonymous Variant<br>NM_000083.3:c.261C>T,<br>NP_000074.3:p.Thr87=	2	NDM1 - CC<br>NDM15 - CC	C (0.66557)	Unknown
NDM1	Rs10282312	Missense Variant<br>NM_000083.3:c.352G>T,<br>NP_000074.3:p.Gly118Trp	3	NDM1 - TT	G (0.02271)	Modify protein function
NDM1	Rs756454039	Intron Variant<br>NM_000083.3:c.980-12T>C	9	NDM1 - TT	T (0.99998)	Alter spliced isoforms ratio
NDM1<br>NDM15	Rs2272252	Intron Variant<br>NM_000083.3:c.1402-9C>T	13	NDM1 - CC<br>NDM15 - CC	C (0.57809)	Alter spliced isoforms ratio
NDM1<br>NDM15	Rs10952548	Intron Variant<br>NM_000083.3:c.1931-62G>A	17	NDM1 - GG<br>NDM15 - GG	G (0.79355)	Unknown
NDM1<br>NDM15	Rs2272251	Synonymous Variant<br>NM_000083.3:c.2154C>T<br>NP_000074.3:p.Asp718=	17	NDM1 - TT<br>NDM15 - TT	C (0.54573)	Unknown
NDM1<br>NDM15	Rs13438232	Missense Variant<br>NM_000083.3:c.2180C>T<br>NP_000074.3:p.Pro727Leu	18	NDM1 - CC<br>NDM15 - CC	C (0.58447)	Modify protein function

Source = NCBI, gnomAD, dbSNP

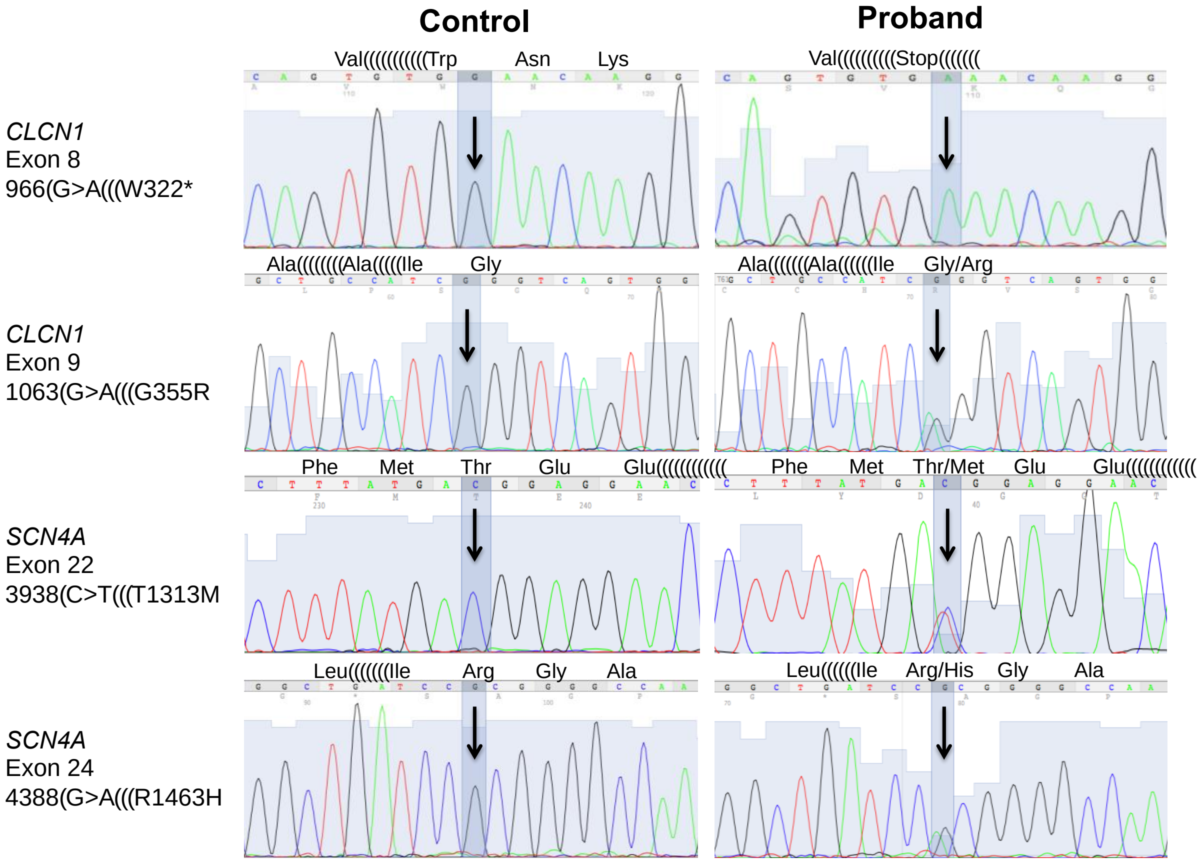

Figure S1. Sanger sequencing showing the mutations found. Electropherograms correspond to the analysis of each proband on each family (see text). Top two panels correspond to the sequencing of exons 8 and 9 of *CLCN1* gene, respectively, and bottom two panels correspond to the sequencing of exons 22 and 24 of *SCN4A* gene, respectively. NDM patients on the right hand and controls on the left-hand side of the figure. The arrows indicate the nucleotide change on each exon.

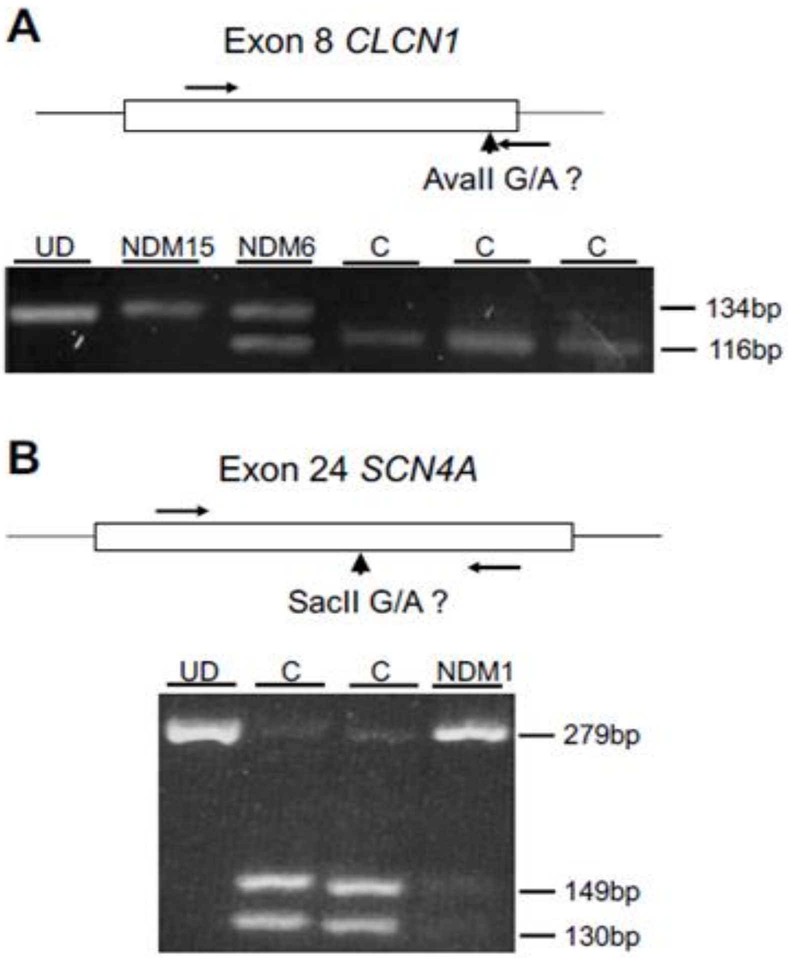

Figure S2. Genotyping assay for the two new mutations found. A. Genotyping of the *CLCN1* gene mutation, W322*. (A) After a PCR using a restriction enzyme-specific primer, PCR products were digested with AvaII. The new variant eliminates the AvaII restriction site, therefore, individuals with the mutation would show at least one undigested product. Top panel shows the position of primers and restriction site. Gel image shows the genotyping of two family members carrying the new mutation (NDM15-homozygous, NDM6-heterozygous) and three controls. The sizes of the digested and undigested PCR products are indicated on the right. UD= AvaII untreated PCR product. **(B)** Genotyping of the *SCN4A* gene mutation, R1463H: After the PCR, PCR products were digested with SacII. The new variant eliminates the SacII restriction site, therefore, individuals with the mutation would show at least one undigested product. Top panel shows the position of primers and restriction site. Gel image shows the genotyping of one carrier of the new mutation (NDM1-heterozygous) and two controls. The sizes of the digested and undigested PCR products are indicated on the right. UD= SacII untreated PCR product.

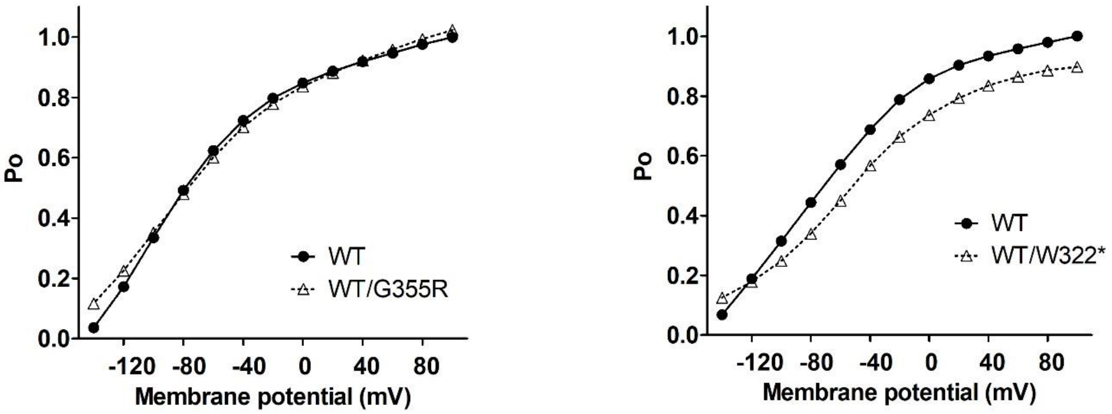

Figure S3. Open probability (Po) of the heterozygous oocytes. The open probability was estimated on the oocytes co-expressing the mutant channel G355R (left) or W322* (right) with the WT channel in a heterozygous state. n = 12 for WT/G355R, n = 13 for WT/W322*, and n = 11 for WT.

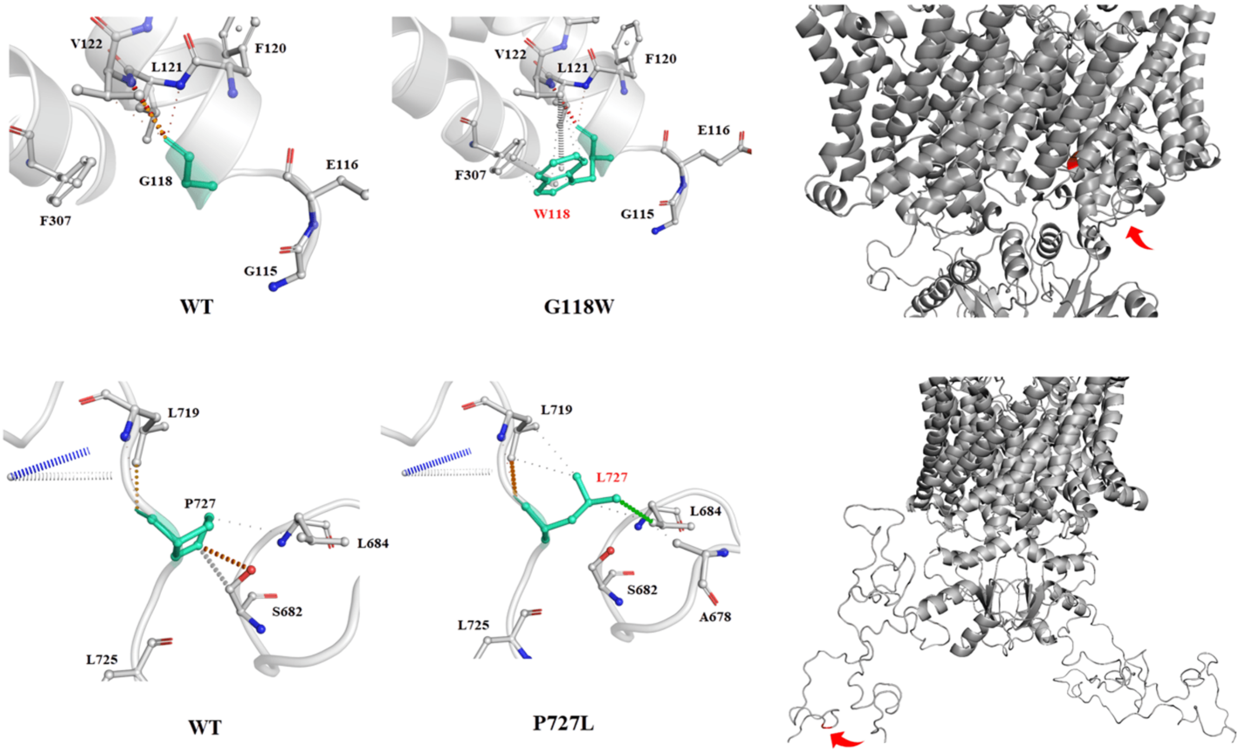

Figure S4. Structural variations derived from G118W and P727L variants in the ClC-1 channel. In general, G118W (top) and P727L (bottom) variants tend to stabilize the region due to novel intermolecular interactions. The residues involved are indicated by the red arrows in the structures (top/bottom right) to locate the variant in the context of the whole channel. It should be expected that these variants do not affect the structure and function of the ClC-1 channel.

References

1. Morales F, Cuenca P, Del Valle G, Brian R, Sittenfeld M, Montoya O, et al. Miotonía congénita: caracterización clínica de una familia costarricense afectada por la Enfermedad de Thomsen. Neuroeje. 2003;17:82-86.
2. Lehmann-Horn F, Mailander V, Heine R, George AL. Myotonia levior is a chloride channel disorder. Hum Mol Genet. 1995;4:1397-402.
